# Increased insular functional connectivity during repetitive negative thinking in major depression and healthy volunteers

**DOI:** 10.1017/S0033291725100925

**Published:** 2025-09-12

**Authors:** Landon S. Edwards, Saampras Ganesan, Jolene Tay, Eli S. Elliott, Masaya Misaki, Evan J. White, Martin P. Paulus, Salvador M. Guinjoan, Aki Tsuchiyagaito

**Affiliations:** 1 Laureate Institute for Brain Research, Tulsa, OK, USA; 2 Department of Biomedical Engineering, The University of Melbourne, Carlton, VIC, Australia; 3 Contemplative Studies Centre, Melbourne School of Psychological Sciences, The University of Melbourne, Melbourne, VIC, Australia; 4 Oxley College of Health and Natural Sciences, The University of Tulsa, Tulsa, OK, USA; 5 Department of Psychiatry, Oklahoma University Health Sciences Center at Tulsa, Tulsa, OK, USA; 6 Laureate Psychiatric Hospital and Clinic, Tulsa, OK, USA; 7 Research Center for Child Mental Development, Chiba University, Chiba, Japan

**Keywords:** depression, functional connectivity, insula, repetitive negative thinking, rumination, resting-state, fMRI

## Abstract

**Background.:**

Repetitive negative thinking (RNT) in major depressive disorder (MDD) involves a persistent focus on negative self-related experiences. Resting-state fMRI shows that the functional connectivity (FC) between the anterior insula and the superior temporal sulcus is associated with RNT intensity. This study examines how insular FC patterns differ between resting state and RNT induction in MDD and healthy control (HC) participants.

**Methods.:**

Forty-one individuals with MDD and 28 HCs (total *n* = 69) underwent resting-state and RNT-induction fMRI scans. Seed-to-whole brain analysis using insular subregions as seeds was performed.

**Results.:**

No diagnosis-by-run interaction effects were observed across insular subregions. MDD participants showed greater FC between the bilateral anterior, middle, and posterior insular regions and the cerebellum (*z* = 4.31–6.15). During RNT induction, both MDD and HC participants demonstrated increased FC between bilateral anterior/middle insula and prefrontal cortices, parietal lobes, posterior cingulate cortex (PCC), and medial temporal gyrus, encompassing the STS (*z* = 4.47–8.31). In exploratory correlation analyses, higher trait RNT was associated with increased FC between the right dorsal anterior/middle insula and the PCC, middle temporal gyrus, and orbital frontal gyrus in MDD participants (*z* = 4.31–6.15). Greater state RNT was linked to increased FC in similar insular regions, as well as the bilateral angular gyrus and right middle temporal gyrus (*z* = 4.47–8.31).

**Conclusions.:**

Hyperconnectivity in insula subregions during active rumination, especially involving the default mode network and salience network, supports theories of heightened self-focused and negative emotional processing in depression. These findings emphasize the neural basis of RNT when actively elicited in MDD.

## Introduction

Repetitive negative thinking (RNT), such as rumination in the context of depression, is a cognitive process characterized by a persistent focus on negative experiences related to the self (Nolen-Hoeksema, Wisco, & Lyubomirsky, 2008). RNT is a symptom dimension with significant implications for the course and prognosis of depression, making this disorder refractory to treatment, chronic, and complicated with suicide (Krajniak, Miranda, & Wheeler, 2013; Surrence, Miranda, Marroquin, & Chan, 2009; Watkins & Roberts, 2020). Previous research has examined the triggers, intensity, and duration of RNT. Characterizing the neurobiological mechanisms of RNT is important not only for understanding its formation but also for identifying neuromodulation targets aimed at alleviating this symptom.

Prior functional connectivity (FC)-based studies have identified many regions of interest (ROIs) as they relate to heightened RNT and brooding symptoms in individuals, including the left dorsolateral prefrontal cortex, precuneus, and other components of the default mode network (DMN) (Jacob et al., 2020; Taylor et al., 2022). However, our previous resting-state fMRI study revealed that RNT intensity correlates with increased FC between the bilateral anterior insular cortices and the right superior temporal sulcus (STS) (Tsuchiyagaito et al., 2022). This result highlighted the neural mechanisms underlying RNT as difficulties in disengaging attention from negative emotional responses (Craig, 2009), and having interrelation with inner-speech processing (Deen, Koldewyn, Kanwisher, & Saxe, 2015). This is compatible with the view that the DMN serves resting self-dialogue, but not necessarily depressive rumination (Goldstein-Piekarski et al., 2022). Thus, prior evidence deemphasizes the role of DMN dysfunction in RNT (Goldstein-Piekarski et al., 2022; Makovac et al., 2020; Tozzi et al., 2021), while recent work by our group (Tsuchiyagaito et al., 2022) demonstrates that the functional connection between the insula (Craig, 2009) and the STS (Deen et al., 2015) is related to the intensity of RNT (Tsuchiyagaito et al., 2022). Nevertheless, our understanding is limited to the resting-state data, which lack clarity on the RNT circuit when individuals are actively engaging with RNT.

RNT has been established as a trait-like cognitive process that involves a recurrent and continuous focus on self-relevant negative thoughts that are persistent over time and across situations. However, RNT intensity can also fluctuate, such that there is a state component to it; it can be influenced by overall depression symptom severity, instant mood state, and adverse environmental stimuli – including relevant interpersonal interactions (Chang et al., 2023; Philippi et al., 2022). This differentiation aligns with recent studies utilizing the experimental induction of RNT, which demonstrates the potential independence and distinct characteristics of both trait RNT and state RNT (Grant, Mills, Judah, & White, 2021; LeMoult, Arditte, D’Avanzato, & Joormann, 2013; Robinson & Alloy, 2003; Wang, Song, Lee, & Zhang, 2022). For example, Misaki et al. (2023) highlighted that while RSFC alterations distinguish between healthy and depressed individuals, trait RNT in depressed individuals is more closely predicted by FC during an induced RNT scan rather than a resting-state scan, suggesting that RNT involves an active mental process not fully represented in the resting-state. While trait RNT measures an individual’s tendency to engage in RNT, induced RNT (capturing instant symptomatology) enables us to probe specific triggers, response patterns, and the phenomenological characteristics of RNT that are not captured by trait RNT alone. Thus, discerning the brain mechanisms that underlie both the trait and state aspects of RNT could have significant implications for clinical practice in terms of RNT remediation.

Given the results of our previous resting-state FC investigations and the prior literature, we aimed to further clarify the mechanistic basis of RNT by comparing insular FC during RNT induction with resting-state FC in individuals with major depressive disorder (MDD). Specifically, we employed a seed-to-whole-brain analysis using six insula subregions as seeds. We hypothesized that individuals with MDD would exhibit a more substantial increase in insular FC during RNT induction compared to resting state, with these alterations being more pronounced in the MDD individuals than in healthy controls (HCs). By investigating these neural dynamics, we seek to address the question: How do the FC patterns of the insula differ between resting state and RNT induction in MDD, and what implications do these differences have for the development of targeted neuromodulatory interventions?

## Methods

### Study design

The study protocol was reviewed and approved by the WCG IRB (https://www.wcgirb.com) (IRB Tracking No. 20210286) and registered on ClinicalTrials.gov (NCT04941066) as a part of a real-time fMRI-neurofeedback (rtfMRI-nf) study (Tsuchiyagaito et al., 2021, 2023b).

### Participants

Forty-one MDD and 28 HC volunteers were recruited for rtfMRI-nf studies, making up a total of 69 participants (Tsuchiyagaito et al., 2021, 2023b). Participants were of both sexes, between the ages of 18 and 65 years, and fluent in English. Exclusion criteria were pregnancy, an abnormal neuromorphological brain profile as assessed by a radiology specialist physician, and other general contraindications for MRI safety. HC participants were defined based on the Mini-International Neuropsychiatric Interview 7.0.2 (MINI) (Sheehan et al., 1998) and confirmed in a clinical conference with a board-certified psychiatrist. MDD-specific inclusion criteria included meeting the criteria of the 5th edition of the *Diagnostic and Statistical Manual of Mental Disorders* (DSM-5) for unipolar MDD based on the MINI (Sheehan et al., 1998) and exhibiting current depressive symptoms with a Montgomery–Åsberg Depression Rating Scale (MADRS) score of >6 (Montgomery & Asberg, 1979). MDD-specific exclusion criteria were as follows: a lifetime history of bipolar disorder, schizophrenia, or any psychotic disorders; DSM-5 criteria for substance abuse or dependence within 6 months prior to study entry; active suicidal ideation as indicated by the Columbia-Suicide Severity Rating Scale (C-SSRS) (Posner et al., 2011) or an attempt within 12 months prior to study entry; commencement of psychotropic medication for depression and/or anxiety less than 1 month before the study enrollment; and commencement of psychological therapy less than 1 month before the study enrollment. All participants completed a written informed consent process before participating in the study.

### Neuroimaging data acquisition

Neuroimaging was conducted on a 3 Tesla MR750 Discovery scanner (GE Healthcare, Milwaukee, WI) with an eight-channel, receive-only head array coil. Blood-oxygen-level-dependent fMRI data were acquired using a T2*-weighted gradient echo-planar sequence with sensitivity encoding (SENSE) with the following parameters: TR/TE = 2,000/25 ms; acquisition matrix = 96 × 96; FOV/slice = 240/2.9 mm; flip angle = 90°; voxel size = 2.5 × 2.5 × 2.9 mm^3^; 40 axial slices; and SENSE acceleration *R* = 2. To provide an anatomical reference for fMRI data, T1-weighted (T1w) MRI images were acquired with a magnetization-prepared rapid gradient-echo sequence with the parameters of FOV = 240 × 192 mm, matrix = 256 × 256, 124 axial slices, slice thickness = 1.2 mm, 0.94 × 0.94 × 1.2 mm^3^ voxel volume, TR/TE = 5/2 ms, SENSE acceleration *R* = 2, flip angle = 8°, delay/inversion time TD/TI = 1,400/725 ms, sampling bandwidth = 31.2 kHz, and scan time = 4 min 59 s.

### Experimentally induced RNT and resting-state scanning

The MRI session started with a 5-min T1w MRI anatomical scan, a 6-min 50-s resting-state fMRI scan, and a 6-min 50-s experimentally induced RNT fMRI scan. Prior to the MRI session, participants identified a recent personal event that significantly triggered RNT, such as experiencing rejection by someone important to them. Participants provided a brief title for this event, which was used by research staff to prompt the participant’s recall immediately before the RNT-inducing fMRI scan. Participants were then instructed about the neurofeedback task as described in detail in Tsuchiyagaito et al. (2021), (2023b), and then had a rest period before the MRI session. In the scanner, the session began with a resting-state scan, where participants were instructed to clear their minds and not think of anything while viewing a fixation cross. This instruction was used intentionally to minimize engagement in spontaneous self-referential or ruminative thoughts, which could confound comparisons with the subsequent RNT-induction scan. After the resting-state scan, participants were reminded of their selected RNT event and instructed to focus on their emotional reactions to it and to reflect on why they responded the way they did – thus entering an RNT state during the scan. While keeping their gaze on the fixation cross, they were asked to focus on their emotional reactions to their chosen event and why they responded the way they did. This procedure aimed to engage the participants in a state of rumination and brooding, characteristic of RNT, while inside the scanner. The MRI session ended with neurofeedback scans as described elsewhere (Tsuchiyagaito et al., 2021, 2023b).

### Symptom measures

#### Trait-RNT

The 22-item Ruminative Response Scale (RRS) (Nolen-Hoeksema & Morrow, 1991) was used to measure trait RNT. The RRS is composed of three subscales: the 5-item ‘brooding’ subscale (e.g. RRS-B item: *think* ‘*why can*’*t I handle things better*’), the 12-item ‘depressive rumination’ subscale (e.g. RRS-D item: *think about all of your shortcomings, failings, faults, and mistakes*), and the 5-item ‘reflection’ subscale (e.g. RRS-R item: *write down what you are thinking and analyse it*). It assesses an individual’s tendency or trait to ruminate when they feel sad or are faced with depressive symptoms. Participants are asked to indicate what they ‘generally do when feeling down, sad, or depressed’ using a 4-point Likert scale ranging from 1 (never) to 4 (always), representing the trait tendency. The items in the RRS-B measure how often people engage in RNT, the causes and consequences of RNT, or a passive comparison with unachieved goals – characteristics that are found to lead to worse prognoses of depression (Treynor, Gonzalez, & Nolen-Hoeksema, 2003). The items in the RRS-D subscale are similar to the RRS-B subscale; however, this subscale measures how often people engage in RNT, with a focus on depressive symptoms and moods. We employed the RRS-B and RRS-D subscales for connectivity analyses related to trait RNT as RRS-R does not include pathological elements of RNT and may even reflect protective factors against depression (Treynor et al., 2003).

#### State-RNT

The level of state RNT that immediately followed the state-RNT fMRI scan was assessed with the Visual Analogue Scale. Right after the resting-state and RNT-induction scans, participants used a button box to answer the question, ‘*To what extent did you dwell on negative aspects of yourself?*’. The answers consisted of ratings from 1 (not at all) to 10 (extremely), indicating the intensity of their state RNT during the scan.

#### Severity of depression and anxiety

Individuals with MDD were assessed before the MRI session using MADRS (Montgomery & Asberg, 1979) and the Hamilton Anxiety Scale (HAM-A) (Maier, Buller, Philipp, & Heuser, 1988).

### Preprocessing

Preprocessing of functional images was performed with Analysis of Functional NeuroImages (AFNI) (http://afni.nimh.nih.gov/afni/). The initial three volumes were excluded from the analysis. The preprocessing included despiking, RETROICOR (Glover, Li, & Ress, 2000), respiratory volume per time (Birn, Smith, Jones, & Bandettini, 2008) physiological noise corrections, slice-timing correction, motion corrections, nonlinear warping to the MNI template brain with resampling to 2 mm^3^ voxels using the Advanced Normalization Tools (Avants, Epstein, Grossman, & Gee, 2008) (http://stnava.github.io/ANTs/), smoothing with 6-mm FWHM kernel, and scaling to percent change relative to the mean signal in each voxel. We used FastSurfer (https://www.sciencedirect.com/science/article/pii/S1053811920304985) to extract white matter and ventricle masks from the anatomical image of an individual subject and then warped them to the normalized fMRI image space. General linear model (GLM) analysis was performed with regressors of 12 motion parameters (three rotations, three shifts, and their temporal derivatives), three principal components of ventricle signals, local white matter average signals (ANATICOR; Jo et al., 2010), fourth-order Legendre polynomials for high-pass filtering, and censoring TRs with large head motion (>0.25-mm frame-wise displacement). Any data with more than 30% censored volumes were treated as a missing value for the group-level analysis (two datasets of HC during RNT induction and two datasets of MDD participants during RNT-induction and resting-state scans were treated as missing values). Voxel-wise residual signals of the GLM were used for the seed-to-whole brain analysis.

### Seed-to-whole brain analysis

#### Definition of insular subregions

In order to better delineate the specific function of the insula, the Brainnetome insula sub-regions parcellation atlas was used (Fan et al., 2016). This parcellation atlas defined fine-grained insular subregions using probabilistic connectivity patterns. The insula was segmented into six subregions in each hemisphere, including the hypergranular insula (G), ventral agranular insula (vla), dorsal agranular insula (Carney et al., 2001; Sliz & Hayley, 2012), ventral dysgranular and granular insula (vId/vIg), dorsal granular insula (dIg), and dorsal dysgranular insula (dId) (Supplementary Figure S1).

#### FC processing

Twelve seed-to-whole brain FC maps were calculated based on predefined insular subregions. The average time course was obtained from the seeds, and the FC maps were generated by calculating Pearson’s correlation coefficients between the time series within the seed and the time series from every other voxel across the whole brain. Correlation coefficients were converted to *z*-scores using Fisher’s *r*-to-*z* transformation.

#### Statistical analysis

AFNI’s 3dLMEr was performed on each seed to identify the connectivity patterns of the insular subregions with the interaction of diagnosis (MDD vs. HC) by run (RNT induction vs. Rest), age, sex, motion, and medication status as fixed effects, and subjects as random intercepts. Head motion was calculated using the six standard motion parameters (three translational and three rotational), and the mean framewise displacement for each scan was included as a covariate in the Linear Mixed Effects Modeling (LME modeling). The results of the main interaction effect, the main effect of diagnosis, and the main effect of run were reported as a chi-square statistic, and post hoc general linear *t*-style tests were specified in case of the significant main effect, as per the output of AFNI’s 3dLMEr. The significant threshold was set as peak *p* < 0.001 and cluster-wise *p* < 0.05/12 (Bonferroni-corrected). AFNI’s 3dClustSim with 10,000 permutation tests was employed to define the cluster-size thresholds (*k* > 143 voxels). Furthermore, exploratory correlation analyses were performed to investigate the association between changes in FC values during RNT-induction scans (relative to resting state), and both trait RNT and Δstate RNT (i.e. the difference between RNT induction and baseline resting state) in the MDD and HC groups, respectively. Average FC values were extracted from significant clusters identified in the main effect contrasts (RNT induction > Rest) for each seed region. These cluster-level values were then used in linear correlation analyses. An uncorrected threshold of *p* < 0.05 was applied, and the results were presented for exploratory purposes, as no correction for multiple comparisons was performed.

## Results

### Demographic and clinical measures

Table 1 shows the demographic data and clinical characteristics of the MDD (*n* = 41) and HC (*n* = 28) participants (total *n* = 69). The majority of these participants were Female and White, and over half of the MDD participants experienced anxiety disorder comorbidities (51.2%) and were treated with antidepressants (51.2%) (Table 1).

### Insular-to-whole brain FC patterns

#### Interaction effect of diagnosis-by-run

We first examined the interaction effect of diagnosis (MDD vs. HC) by run (resting state vs. RNT induction). Contrary to our hypothesis, no significant FC alterations were observed for the diagnosis-by-run interaction across any of the insular subregions. Results with a threshold of *p* < 0.001, without cluster thresholding, are presented in Supplementary Figures S2 and S3.

#### Main effect of diagnosis and run

Participants with MDD demonstrated greater FC between the bilateral anterior, middle, and posterior insular regions and the cerebellum (*z* = 4.31–6.15). These results suggest a unique pattern of insular-cerebellar connectivity in MDD (Table 2 and Supplementary Figures S4 and S5).

**Figure 1.**
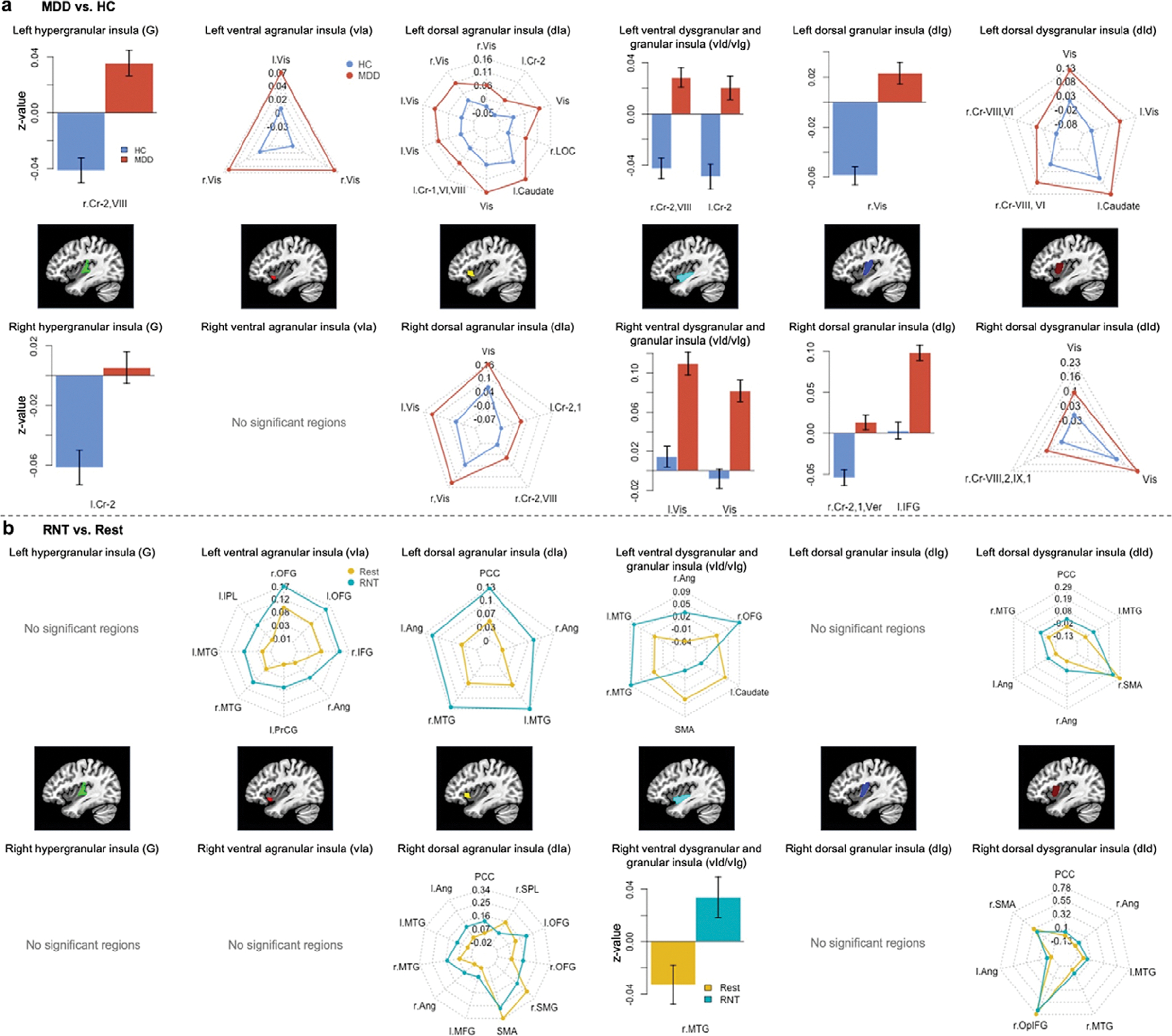
Post hoc investigation of (a) the effect of diagnosis (major depressive disorder vs. healthy control participants) and (b) the effect of run (RNT induction vs. Rest). Abbreviations: L, left; R, right; Cr, cerebellum; Vis, visual area; Ver, vermis; IFG, inferior frontal gyrus; OFG, orbital frontal gyrus; Ang, angular gyrus; PrCG, precentral gyrus; MTG, middle temporal gyrus; IPL, inferior parietal lobule; PCC, posterior cingulate cortex; SMG, supramarginal gyrus; SPL, superior parietal lobule; SMA, supplementary motor area; OpIFG, opercular part of the inferior frontal gyrus; RNT, repetitive negative thinking.

Regarding the main effect of run (Table 3 and Supplementary Figures S6 and S7), enhanced FC was found between the bilateral anterior and middle insula and other key brain regions, including the bilateral prefrontal cortices, parietal lobes, posterior cingulate cortex (PCC), and medial temporal gyrus, encompassing the STS (*z* = 4.47–8.31). Figure 1 displays additional spider charts and bar plots to illustrate the post hoc effects of these main findings.

### Correlation between insular FC and RNT measures

**Figure 2.**
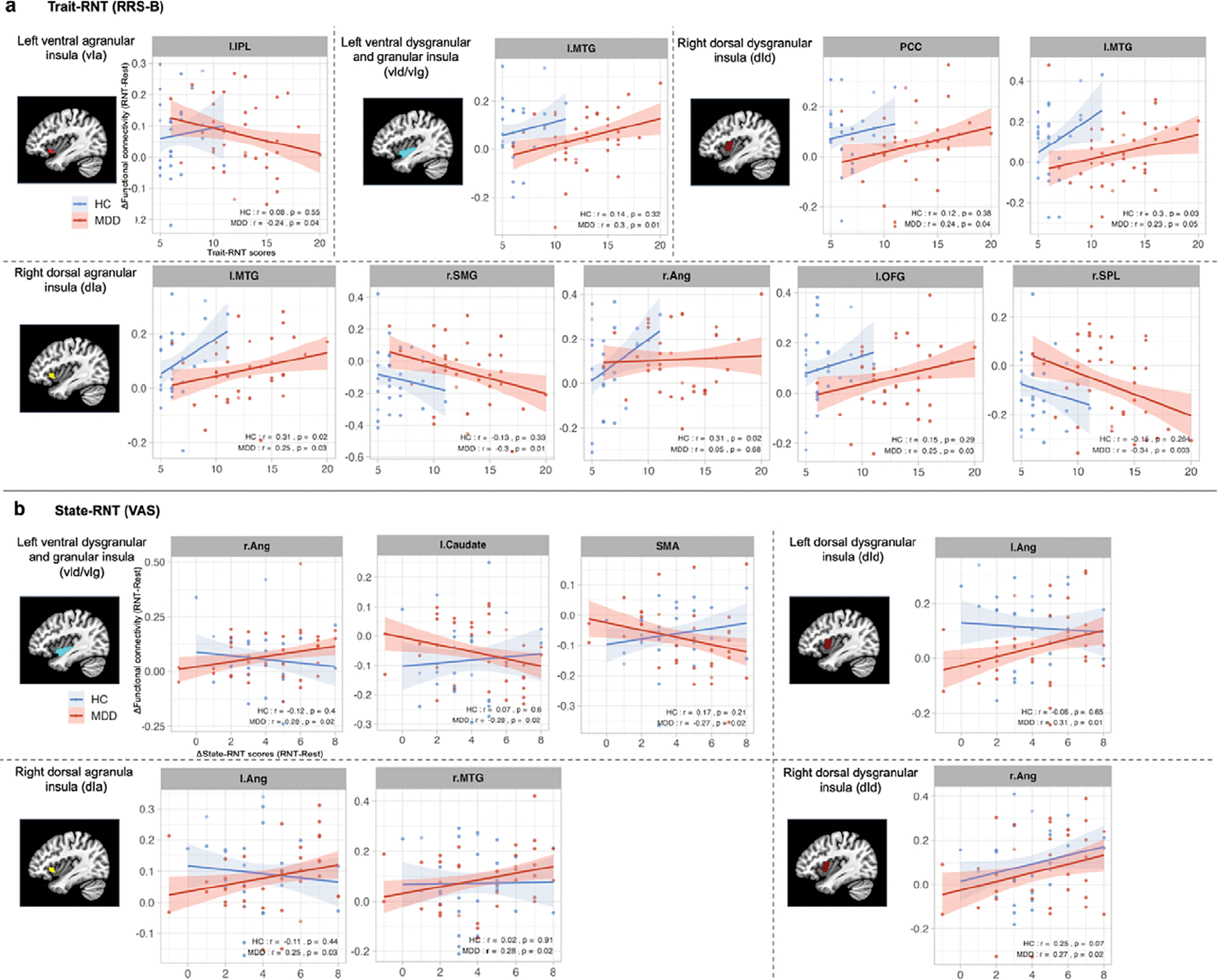
Scatter plots and correlation between insular-cortical functional connectivity (FC) and RNT measures. (a) Correlation of trait RNT as measured by the Ruminative Response Scale-Brooding subscale (RRS-B) before the scan (*x*-axis) with changes in FC during RNT-induction scan compared to the Rest scan (*y*-axis). (b) Correlation of changes in state RNT as measured by the Visual Analogue Scale during RNT-induction scan compared to the Rest scan (*x*-axis) with changes in FC during RNT-induction scan compared to Rest scan (*y*-axis). Abbreviations: L, left; R, right; IPL, inferior parietal lobule; MTG, middle temporal gyrus; PCC, posterior cingulate cortex; SMG, supramarginal gyrus; Ang, angular gyrus; OFG, orbital frontal gyrus; SPL, superior parietal lobule; SMA, supplementary motor area; RNT, repetitive negative thinking.

Figure 2 depicts significant associations between RNT measures and FC of the insular cortex with other regions in MDD participants, as well as HC participants. Consistent with our findings in increased insular FC during RNT induction relative to the resting state, among individuals with MDD, higher trait RNT was positively associated with increased FC between the right dorsal anterior and middle insula, the regions in the DMN (including the PCC and middle temporal gyrus), and the regions in the salience network (SN) (including the orbital frontal gyrus). Moreover, greater state-RNT scores during RNT induction, compared to resting-state, were positively correlated with increased FC in similar insular regions and the bilateral angular gyrus, as well as the right middle temporal gyrus (Figure 2). On the other hand, higher trait RNT was negatively correlated with increased insular FC between the left anterior insula and the inferior parietal lobule in individuals with MDD, although this FC showed an increased main effect of RNT induction (Table 3 and Figure 1).

## Discussion

This study investigated the hypothesis that individuals with MDD would demonstrate a greater increase in FC between the insular cortex and other cortical (including cerebellar) regions during RNT induction compared to resting state. We also predicted that functional changes would be more pronounced in MDD, as compared with HC individuals. We observed three main findings during our research by which our hypothesis was partially supported. First, contrary to our hypothesis, there was no statistically significant diagnosis-by-run interaction in insular FC, indicating that changes in FC during RNT induction are not significantly different in individuals with MDD compared to HC individuals. Second, FC between insular and cerebellar cortices was higher in individuals with MDD compared to the HC group. Third, overall, FC between insular and other cortical regions increased during RNT induction compared to resting-state data.

Altogether, these findings support the hypothesis that the visceral control and higher-order cognitive processing changes underlie RNT intensity (Tsuchiyagaito et al., 2022). These findings also reflect that insular-cortical FC was stronger during RNT induction compared with resting state. However, our results did not demonstrate a significant difference in FC alterations during RNT induction between the MDD and HC participants.

### Insular connectivity in MDD

The observed higher FC between the anterior, middle, and posterior insula and the cerebellum in MDD participants aligns with emerging literature implicating cerebellar contributions to emotional and cognitive processes in depression (Depping, Schmitgen, Kubera, & Wolf, 2018; Habas, 2021; Misaki et al., 2023; Pierce et al., 2023; Sliz & Hayley, 2012; Van Overwalle et al., 2020). In our study, significant clusters were located in Crus I, Crus II, lobule VI, lobule VIII, and lobule IX, with bilateral involvement and some extension into the vermis. These regions have been associated with non-motor functions, including emotion regulation, self-referential processing, working memory, and affective appraisal (Buckner et al., 2011; Depping et al., 2018; Stoodley & Schmahmann, 2009). For instance, Crus I and Crus II show intrinsic connectivity with prefrontal and parietal cortices and are considered part of a cognitive-affective cerebellar network (Depping et al., 2018; Pierce et al., 2023). Lobule VI serves as a transitional zone between motor and association regions, contributing to both sensorimotor control and emotional monitoring (Depping et al., 2018; Schmahmann, 2004). Additionally, lobule VIII and lobuleIX, though traditionally linked to sensorimotor functions, are increasingly recognized for their role in affective and visceral regulation, particularly in conjunction with the insula (Pierce et al., 2023). These findings suggest that increased insula–cerebellar FC observed in MDD may reflect dysregulation in circuits underlying interoceptive awareness, emotional salience detection, and self-focused negative appraisal, all of which are hallmarks of RNT (Depping et al., 2018; Habas, 2021; Misaki et al., 2023; Pierce et al., 2023; Sliz& Hayley, 2012; Van Overwalle et al., 2020). These findings support the notion that cerebellar subregions interact with cortical salience and DMNs during affective processing (Prati, Pontes-Silva, & Gianlorenço, 2024), and may contribute to the persistent and ruminative symptomatology of depression. Future studies employing cerebellar parcellation and task-based designs may further clarify the role of these distinct subregions in the pathophysiology of MDD.

The insula participates in a multitude of functions related to information processing and emotional feeling via its connections to the limbic and autonomic nervous systems (Gasquoine, 2014). In addition, prior neuroimaging studies have noted insula hyperactivation and increased blood flow and metabolism in patients with major depression and other mental disorders (Gasquoine, 2014). Given this, it is possible that the insula contributes to the negative symptomology experienced during RNT due to its role in interpreting interoceptive and perceptual data. Furthermore, known for integrating somatosensory, affective, and cognitive information (Sliz & Hayley, 2012), the insula may be crucial in maintaining the heightened state of the negative self-focus aspect of RNT. Prior neuroimaging research has associated emotional recalling/remembrance and other cognitively demanding, emotional tasks with increased insular activity (Phan, Wager, Taylor, & Liberzon, 2002). In this context, our observation of increased FC between the insula and other brain regions during RNT-induction tasks is not surprising. Furthermore, this suggests that the trained regulation of insular activity, or decreasing FC between the bilateral insula and other areas such as the STS, the parietal cortices, or the PCC, may reduce state-RNT symptoms. For example, the emergence of focused deep brain neuromodulation or real-time fMRI neurofeedback in FC literature prompts deeper exploration of these brain activity regulation methods as a means to improve RNT in depression.

The cerebellum’s role in cognitive and emotional processing, traditionally recognized in the last two decades (Pierce et al., 2023; Rudolph et al., 2023), appears to be particularly significant in the context of mood disorders. Previous resting-state static and dynamic FC research identifies the cerebellum as having a vital role in emotional processing and executive functioning through its connections to the executive network, the DMN, the SN, the insula, and multiple brain cortical hubs associated with emotion regulation (Habas, 2021). These connections suggest that the cerebellum is involved in frequent, multimodal collaboration with other crucial brain regions and networks to undertake the multifaceted nature of human emotions. Considering the cerebellum’s association with emotion regulation and other key networks, the cerebellum may also be an ROI worth investigating in future FC studies involving clinically depressed populations.

### Alterations during RNT induction

The augmentation of FC during RNT induction between insular regions and areas, such as the prefrontal and parietal cortices, PCC, medial temporal gyrus, and the STS, is particularly noteworthy. These regions are implicated in a wide range of processes, from self-referential thought to emotional processing and memory retrieval. The increased connectivity that was noticed during RNT induction in our work suggests a heightened state of neural coordination in these networks, potentially underpinning the ruminative process. However, one key limitation to interpreting the relationship between RNT induction and the observed increase in insular connectivity stems from the absence of another specific comparator condition with which we could compare connectivity patterns among rest, RNT induction, and other instructed cognitive engagement (Pillet, Op de Beeck, & Lee Masson, 2020). While the current findings highlight differences between RNT and rest, it is difficult to deduce strong conclusions about the neural activity that is unique to RNT and that which is distinctive of any other type of instructed cognitive activity.

Foregoing studies have presented similar results, demonstrating significant transformations in FC that occur in state RNT. In another mood-induction study, researchers found that increased connectivity between the DMN and the fronto-parietal network (FPN), along with decreased connectivity between the SN and the FPN, are both associated with increased RNT after experiencing sadness (Lydon-Staley et al., 2019). The changes in RNT-induced FC that were observed during our research, particularly with the MDD sample population, were congruent to the findings of their research. In these types of RNT-induction studies, a variety of key networks and brain regions can be observed at play in emotion regulation, many of which may serve as potential targets for interventions and future research aimed at reducing trait- and/or state-RNT symptoms.

### Correlation between insular FC and trait- and state-RNT scores

The correlation of both trait- and state-RNT scores with increased FC in specific brain regions, particularly in MDD patients as reported herein, suggests that FC could be a potential biomarker for RNT severity in clinical settings. Specifically, trait-RNT scores were associated with the increased insular FC of several key regions in the DMN and orbitofrontal gyrus, which are implicated in self-referential and emotional processing (Northoff et al., 2006; Rempel-Clower, 2007). This association highlights the neural correlates of a general propensity to engage in RNT, reflecting a stable, trait-like aspect of cognitive processing in individuals. In contrast, state-RNT scores were associated with FC between the insula and the angular gyrus, as well as the right medial temporal gyrus, during experimentally induced RNT. Changes in state-RNT ratings indicate how participants engaged with RNT during experimental induction relative to the resting state. The association with increased FC in these regions suggests that the acute induction of RNT may engage neural circuits related to memory, conceptual processing (Deen et al., 2015; Humphreys, Ralph, & Simons, 2021; Ramanan, Piguet, & Irish, 2018; Seghier, 2013), and the integration of emotional and sensory information (Craig, 2009). This distinction underlines the dynamic nature of RNT, where state-dependent increases in RNT were correlated with immediate neural responses, differentiating it from the more static trait RNT. Such findings illustrate the complex neural underpinnings of RNT, supporting the idea that different facets of RNT are potentially supported by different neural networks, as reported in prior studies (Rosenbaum et al., 2017; Tsuchiyagaito et al., 2023a). However, we would caution against any definitive conclusions based on correlation analysis due to the exploratory nature of this analysis.

### Limitations and future directions

While our findings contribute significantly to the understanding of RNT in MDD, several limitations must be acknowledged, including the small sample size and the absence of an additional specific comparator condition. Longitudinal studies, or interventional studies using emerging neuromodulation methods to non-invasively modulate the large-scale circuits described herein (Philip & Arulpragasam, 2023), could help to establish a causative role of neural alterations in RNT. Additionally, incorporating another comparator condition (such as reflecting on a past positive or neutral experience) into the study protocol may help distinguish RNT-induced cognitive activity from resting or other instructed mental states, thereby improving interpretability (Pillet et al., 2020). In the present study, we conducted separate linear mixed-effects models for each insular subregion to maintain analytic clarity and avoid overcomplicating the model structure. However, we acknowledge that an alternative approach, modeling all seed regions within a single LME framework that includes seed region, hemisphere, run, and diagnostic group as factors, could offer a more integrated view of insular connectivity. We did not pursue this strategy due to the analytic complexity of estimating higher-order interactions in a modestly sized sample, which could limit statistical power and affect model stability. Future studies with larger datasets may benefit from implementing such a comprehensive model to test for interaction effects across insular subregions in a unified manner.

Moreover, preceding research by our group and others has suggested that RNT is a transdiagnostic occurrence, as it is a usual feature in individuals with generalized anxiety disorder (GAD) and obsessive–compulsive disorder (OCD) (Wahl et al., 2019). Given the comorbidity of these disorders, it may be worth conducting a similar investigation that explores FC developments and trait/state RNT with participants from GAD and OCD populations.

## Conclusion

The findings of our study underscore the importance of insular connectivity in the neural systems underlying RNT in MDD. Individuals with MDD exhibit distinct FC patterns between the insula and the cerebellum, highlighting a neural circuit that may contribute to the persistence and intensity of RNT. In addition, both MDD and HC participants show increased insular connectivity with key brain regions, including the bilateral prefrontal cortices, parietal lobes, PCC, and medial temporal gyrus, during RNT induction compared to resting state. This suggests that the insula is part of a broader network that becomes more engaged during active RNT, facilitating the integration of emotional and cognitive aspects of negative self-related thoughts. However, it is again important to acknowledge that firm conclusions related to the differences in FC patterns noted between RNT induction and rest cannot be attributed uniquely to RNT due to the lack of an additional comparator condition.

Furthermore, higher trait-RNT in MDD participants was associated with increased connectivity between the insula and the regions within the DMN and the SN, indicating that persistent negative thinking is linked to specific insular connectivity patterns involving self-referential processing and emotional salience. These differential connectivity patterns, including regions where higher trait RNT is negatively correlated with increased insular connectivity, may serve as neural markers for the intensity of RNT. Taken together, our findings highlight an association between insular connectivity and its interactions with other brain regions in the manifestation of RNT, providing a foundation for the development of targeted neuromodulatory interventions to alleviate this symptom in depression. This is in line with emerging neuromodulation techniques with anatomical specificity (Mehić et al., 2014; Siddiqi et al., 2020) that can be used to modulate this circuitry.

## Supplementary Material

Supplementary Material

**Supplementary material.** The supplementary material for this article can be found at https://doi.org/10.1017/S0033291725100925.

## Figures and Tables

**Table 1.** Demographic data

Demographic data	MDD		HC	
	Mean/number	SD/%	Mean/number	SD/%
Age	36.54	(12.27)	23.04	(3.92)
Female (%)	31	(75.61)	21	(75)
Handedness: Right (%)	37	(90.24)	28	(100)
Race/ethnicity: Non-white (%)	8	(19.51)	13	(46.43)
Asian	0	(0)	1	(3.57)
Black/African American	0	(0)	1	(3.57)
Native Hawaiian/Pacific Islander	0	(0)	1	(3.57)
Hispanic	1	(2.44)	7	(25)
Indigenous	7	(17.07)	1	(3.57)
Multiracial	0	(0)	2	(7.14)
White	33	(80.49)	15	(53.57)
Diagnosis (%)				
MDD without comorbidity	20	(48.78)		
MDD and anxiety disorder	21	(51.22)		
Generalized anxiety disorder	13	(31.71)		
Social anxiety disorder	7	(17.07)		
Panic disorder	10	(24.39)		
Depressive episode (%)				
Single episode	14	(34.15)		
Recurrent	27	(65.85)		
Medicated (%)	21	(51.22)		
Antidepressants	18	(43.9)		
Stimulants	5	(12.2)		
Sedatives	7	(17.07)		
Current psychotherapy (%)	9	(21.95)		
**Clinical measures**				
RRS brooding	12.61	(3.13)	6.46	(1.53)
RRS depressive rumination	30.76	(6.76)	15.36	(3.96)
RRS reflection	11.07	(3.34)	6.79	(3.13)
RRS total	54.44	(11.60)	28.61	(7.28)
State RNT (VAS) – Rest	2.59	(2.23)	2.03	(1.28)
State RNT (VAS) – Induction	7.08	(1.66)	5.61	(1.81)
MADRS	19.9	(5.86)		
HAM-A	16.15	(5.22)		

Abbreviations: HAM-A, Hamilton Anxiety Scale; MDRS, Montgomery–Åsberg Depression Rating Scale; MDD, major depressive disorder; RNT, repetitive negative thinking; RRS, Ruminative Response Scale; VAS, Visual Analogue Scale.

**Table 2.** Significant regions showing the main effect of diagnosis (MDD and HC) from seed-to-whole brain functional connectivity analysis

Brain regions	MNI coordinate			Voxels (2 mm^3^)	Chisquare	z-score (MDD – HC)
	x	y	z			
Left insula subregions as seeds						
**1. G-seed**						
Right cerebellum (Crus 2, VIII)	17	−71	−41	416	28.83	5.61
**2. vIa-seed**						
Left visual area	−13	−93	13	290	20.41	4.46
Right visual area	23	−89	9	223	27.40	5.13
Right visual area	31	−69	−19	198	25.97	5.22
**3. dIa-seed**						
Right visual area	19	−81	−39	568	25.08	5.01
Right visual area	19	−87	21	379	26.25	5.30
Left visual area	−23	−77	−17	327	27.48	5.42
Left visual area	−25	−91	17	320	22.78	4.78
Right cerebellum (Crus 1, VI, VIII)	37	−45	−37	197	28.72	5.58
Bilateral visual area	5	−83	45	192	25.99	5.20
Left ventral caudate	−21	27	−7	179	31.26	5.91
Right lateral occipital cortex, middle temporal gyrus	33	−69	19	174	23.32	4.65
Bilateral visual area	7	−53	1	165	24.70	5.05
Left cerebellum (Crus 2)	−15	−87	−39	151	21.15	4.54
**4. vId/vIg-seed**						
Right cerebellum (Crus 2, VIII)	13	−71	−41	304	22.11	4.70
Left cerebellum (Crus 2)	−21	−79	−39	154	25.03	5.10
**5. dId-seed**						
Bilateral visual area	−1	−77	−3	305	25.03	5.09
Right cerebellum (Crus 2, Crus 1, VIII)	23	−77	−39	284	26.50	5.31
Right cerebellum (VIII, VI)	29	−43	−39	179	25.37	5.14
Left ventral caudate	−11	31	−3	165	20.96	4.52
Left visual area	−33	−81	−21	159	20.69	4.49
**6. dIg-seed**						
Right visual area	19	−71	−41	1384	33.36	6.15
**Right insula subregions as seeds**						
**1. G-seed**						
Left cerebellum (Crus 2, VII)	−11	−79	−39	145	23.91	4.93
**2. vIa-seed**						
N/A						
**3. dIa-seed**						
Bilateral visual area	5	−83	43	438	27.35	5.4
Left visual area	−23	−77	−17	349	26.65	5.3
Right visual area	27	−69	1	229	23.74	4.92
Right cerebellum (Crus 2, VIII)	21	−75	−39	186	19.49	4.31
Left cerebellum (Crus 2, Crus 1)	−21	−85	−41	167	25.74	5.16
**4. vId/vIg-seed**						
Left visual area	−25	−75	−19	217	23.94	4.94
Bilateral visual area	15	−77	−13	145	23.39	4.88
**5. dId-seed**						
Bilateral visual area	3	−79	−5	320	22.71	4.78
Right cerebellum (VIII, Crus 2, IX, Crus 1)	19	−73	−41	216	28.28	5.52
Bilateral visual area	1	−65	9	166	21.37	4.58
**6. dIg-seed**						
Right cerebellum (Crus 2, Crus 1, Vermis, VIII)	5	−77	−29	451	25.91	5.21
Left inferior frontal gyrus	−33	7	23	163	23.69	4.92

Abbreviations: HC, healthy control; MDD, major depressive disorder.

**Table 3.** Significant regions showing the main effect of run (RNT-induction and Rest) from seed-to-whole brain functional connectivity analysis

Brain regions	MNI coordinate			Voxels (2 mm^3^)	Chi-square	z-score (RNT – Rest)
	x	y	z			
Left insula subregions as seeds						
**1. G-seed**						
N/A						
**2. vIa-seed**						
Right middle orbital gyrus, inferior frontal gyrus	35	45	−11	525	48.47	8.14
Left inferior parietal lobule	−35	−63	51	435	25.27	5.1
Left middle temporal gyrus	−57	−59	−13	391	32.44	6.05
Right middle temporal gyrus	59	−43	−13	348	28.66	5.57
Left precentral gyrus, inferior frontal gyrus	−45	9	35	213	20.83	4.5
Right angular gyrus, inferior parietal lobule	37	−63	39	198	26.91	5.33
Right inferior frontal gyrus	55	17	25	197	20.64	4.48
Left middle orbital gyrus, inferior frontal gyrus	−43	47	−13	164	26.44	5.27
**3. dIa-seed**						
PCC	5	−39	31	2527	38.62	6.82
Left angular gyrus, superior parietal lobule	−41	−69	53	664	32.59	6.06
Right middle temporal gyrus, STS	63	−33	−9	562	32.75	6.09
Left middle temporal gyrus, STS	−65	−43	−7	548	30.90	5.87
Right angular gyrus, superior parietal lobule	37	−65	43	469	28.81	5.61
**4. vId/vIg-seed**						
Left angular gyrus, superior parietal lobule	37	−71	53	658	32.59	6.03
Left inferior temporal gyrus, middle temporal gyrus	−59	−61	−11	312	37.17	6.66
Right inferior temporal gyrus, middle temporal gyrus	63	−41	−9	302	37.02	6.61
Bilateral supplementary motor area	3	11	67	236	24.08	−4.95
Left caudate, putamen	−17	13	7	181	25.57	−5.15
Right middle orbital gyrus, inferior frontal gyrus	45	47	−19	178	22.21	4.70
**5. dId-seed**						
PCC	−1	−33	41	771	30.35	5.80
Right middle temporal gyrus, STS	65	−37	−9	513	30.80	5.84
Left angular gyrus, inferior parietal lobule	−33	−73	41	487	32.04	6.01
Right angular gyrus, inferior parietal lobule	37	−71	53	465	33.71	6.2
Right supplementary motor area	3	11	59	364	29.89	−5.74
Left middle temporal gyrus, STS	−65	−45	−7	356	33.91	6.23
**6. dIg-seed**						
N/A						
**Right insula subregions as seeds**						
**1. G-seed**						
N/A						
**2. vIa-seed**						
N/A						
**3. dIa-seed**						
PCC	−1	−33	39	2925	49.76	8.31
Left angular gyrus, inferior parietal lobule	−41	−69	53	1477	36.23	6.66
Left middle temporal gyrus, STS	−67	−43	−5	765	30.22	5.77
Right middle temporal gyrus, STS	65	−37	−7	703	38.42	6.83
Right angular gyrus, inferior parietal lobule	39	−65	45	430	26.90	5.36
Left middle frontal gyrus	−25	31	47	362	25.91	5.21
Bilateral supplementary motor area	1	9	61	254	25.95	−5.2
Right supramarginal gyrus	65	−21	43	246	24.15	−4.97
Right middle orbital gyrus, inferior frontal gyrus	39	43	−13	179	20.64	4.47
Left middle orbital gyrus, inferior frontal gyrus	−37	43	−7	175	25.84	5.19
Right superior parietal lobule	19	−57	57	151	20.62	−4.47
**4. vId/vIg-seed**						
Right middle temporal gyrus, inferior temporal gyrus	59	−43	−9	144	25.53	5.13
**5. dId-seed**						
PCC	−3	−33	41	1220	38.86	6.89
Right supplementary motor area	15	9	55	417	34.55	−6.33
Left angular gyrus, inferior parietal lobule	−35	−65	33	368	27.07	5.36
Right inferior frontal gyrus, Rolandic operculum	43	9	3	272	26.27	−5.25
Right middle temporal gyrus, STS, inferior temporal gyrus	65	−17	−21	244	30.21	5.79
Left middle temporal gyrus, STS, inferior temporal gyrus	−65	−45	−7	223	27.50	5.41
Right angular gyrus, superior parietal lobule	37	−67	45	165	22.98	4.8
**6. dIg-seed**						
N/A						

Abbreviations: PCC, posterior cingulate cortex; RNT, repetitive negative thinking; STS, superior temporal sulcus.
