# Supplementary material for: Increased insular functional connectivity during repetitive negative thinking in major depression and healthy volunteers
Source: Psychol Med. 2025 Sep 12;55:e268. doi: 10.1017/S0033291725100925 (PMC12803699; doi:10.1017/S0033291725100925)
Supplement: Edwards et al. supplementary material [file S0033291725100925sup001.pdf]

Supplementary materials for: Increased Insular Functional Connectivity During Repetitive Negative Thinking in Major Depression and Healthy Volunteers

Landon S Edwards[a], Saampras Ganesan[b,c], Jolene Tay[a], Eli S Elliott[a], Masaya Misaki[a,d], Evan J White[a,d], Martin P Paulus[a,d], Salvador M Guinjoan[a,e,f], Aki Tsuchiyagaito[a,d,g]

[a] Laureate Institute for Brain Research, Tulsa, OK, USA
[b] Department of Biomedical Engineering, The University of Melbourne, Carlton, Victoria 3053, Australia
[c] Contemplative Studies Centre, Melbourne School of Psychological Sciences, The University of Melbourne, Melbourne, Victoria 3010, Australia
[d] Oxley College of Health and Natural Sciences, The University of Tulsa, Tulsa, OK, USA
[e] Department of Psychiatry, Oklahoma University Health Sciences Center at Tulsa, Tulsa, OK, USA
[f] Laureate Psychiatric Hospital and Clinic, Tulsa, OK, USA
[g] Research Center for Child Mental Development, Chiba University, Chiba, Japan

[#] Corresponding Author:
Aki Tsuchiyagaito
Laureate Institute for Brain Research
6655 South Yale Ave. Tulsa, OK 74136, USA
Tel: 918-502-5112
E-mail: atsuchiyagaito@laureateinstitute.org

Table of Contents

1. Supplementary figures *3*

Figure S1 3

Figure S2 4

Figure S2 (Continued) 5

Figure S3 6

Figure S3 (Continued) 7

Figure S4 8

Figure S4 (Continued) 9

Figure S5 10

Figure S5 (Continued) 11

Figure S6 12

Figure S6 (Continued) 13

Figure S7 14

Figure S7 (Continued) 15

References 16

1. Supplementary figures

Figure S1

Insula subregions defined based on the Brainnetome atlas.

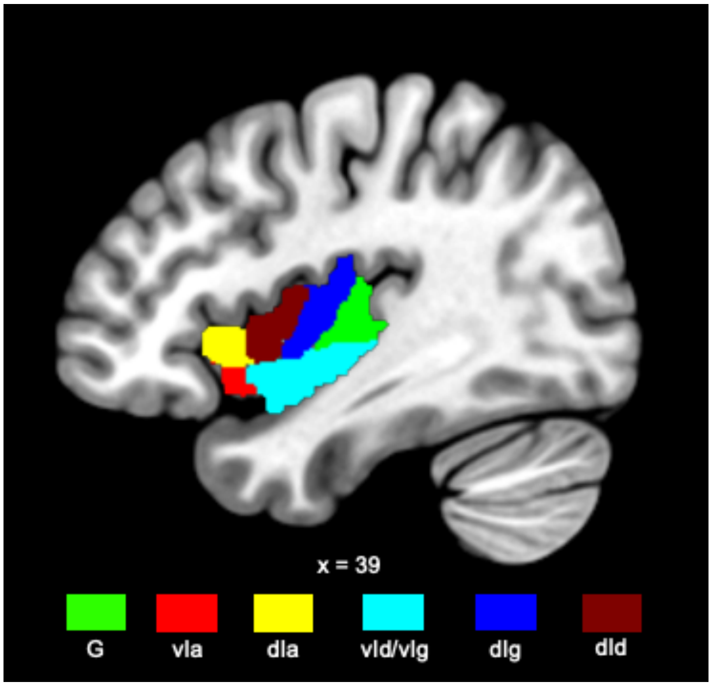

Note. G, hypergranular insula; vIa, ventral agranular insula; dIa, dorsal agranular insula; vId/vIg, ventral dysgranular and granular insula; dIg, dorsal granular insula; dId, dorsal dysgranular insula

Figure S2

Functional connectivity during RNT compared to Rest in individuals with MDD, relative to HC (left insula subregions).

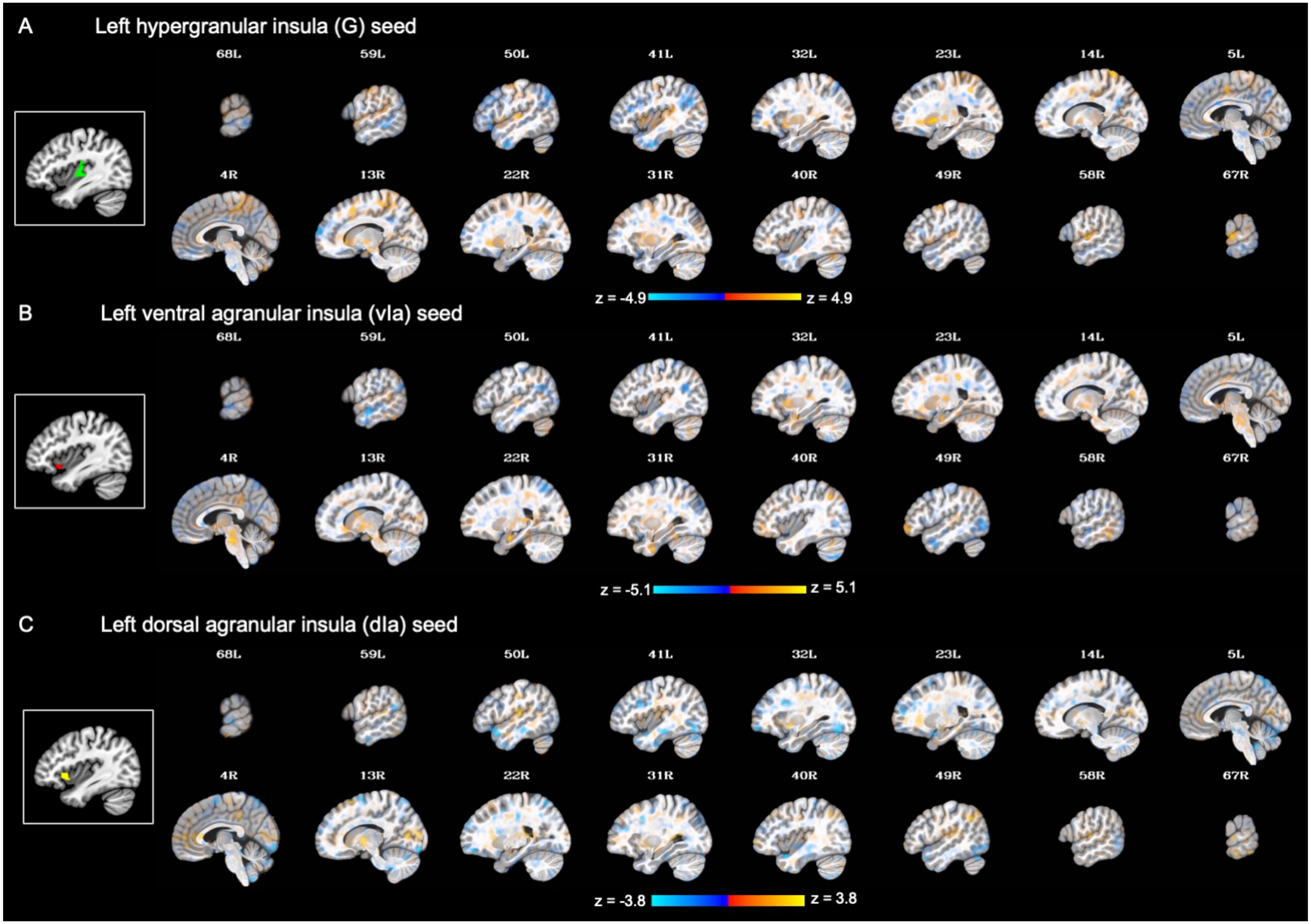

Figure S2 (Continued)

Functional connectivity during RNT compared to Rest in individuals with MDD, relative to HC (left insula subregions).

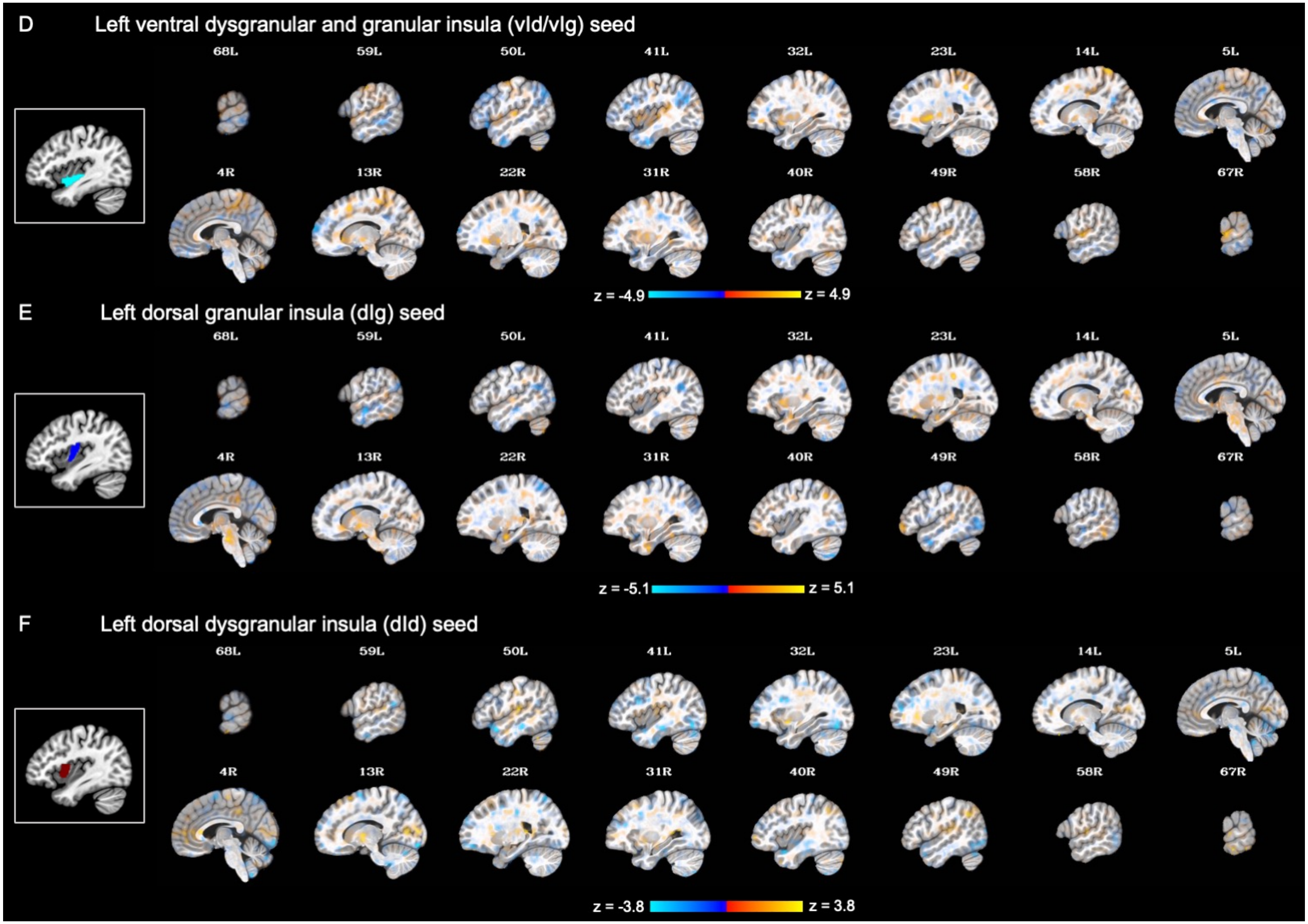

Note. A translucent statistical threshold set at $p < 0.001$ is applied, without cluster-extent correction. Areas exhibit increasing transparency corresponding to decreasing statistical significance (Taylor et al., 2023). Each seed region is displayed on the left side of its corresponding statistical map, framed with a white square outline.

Figure S3

Functional connectivity during RNT compared to Rest in individuals with MDD, relative to HC (right insula subregions).

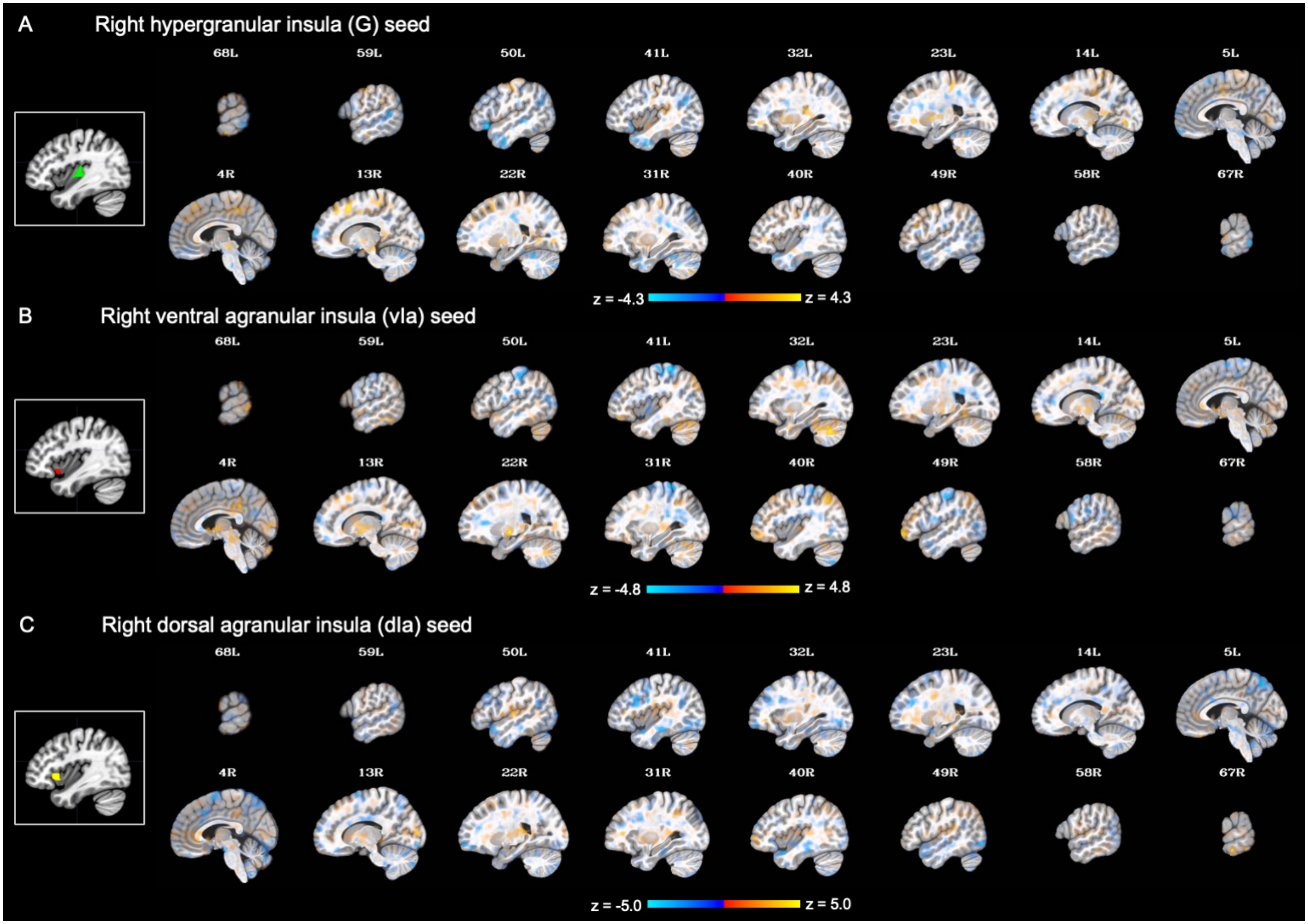

Figure S3 (Continued)

Functional connectivity during RNT compared to Rest in individuals with MDD, relative to HC (right insula subregions).

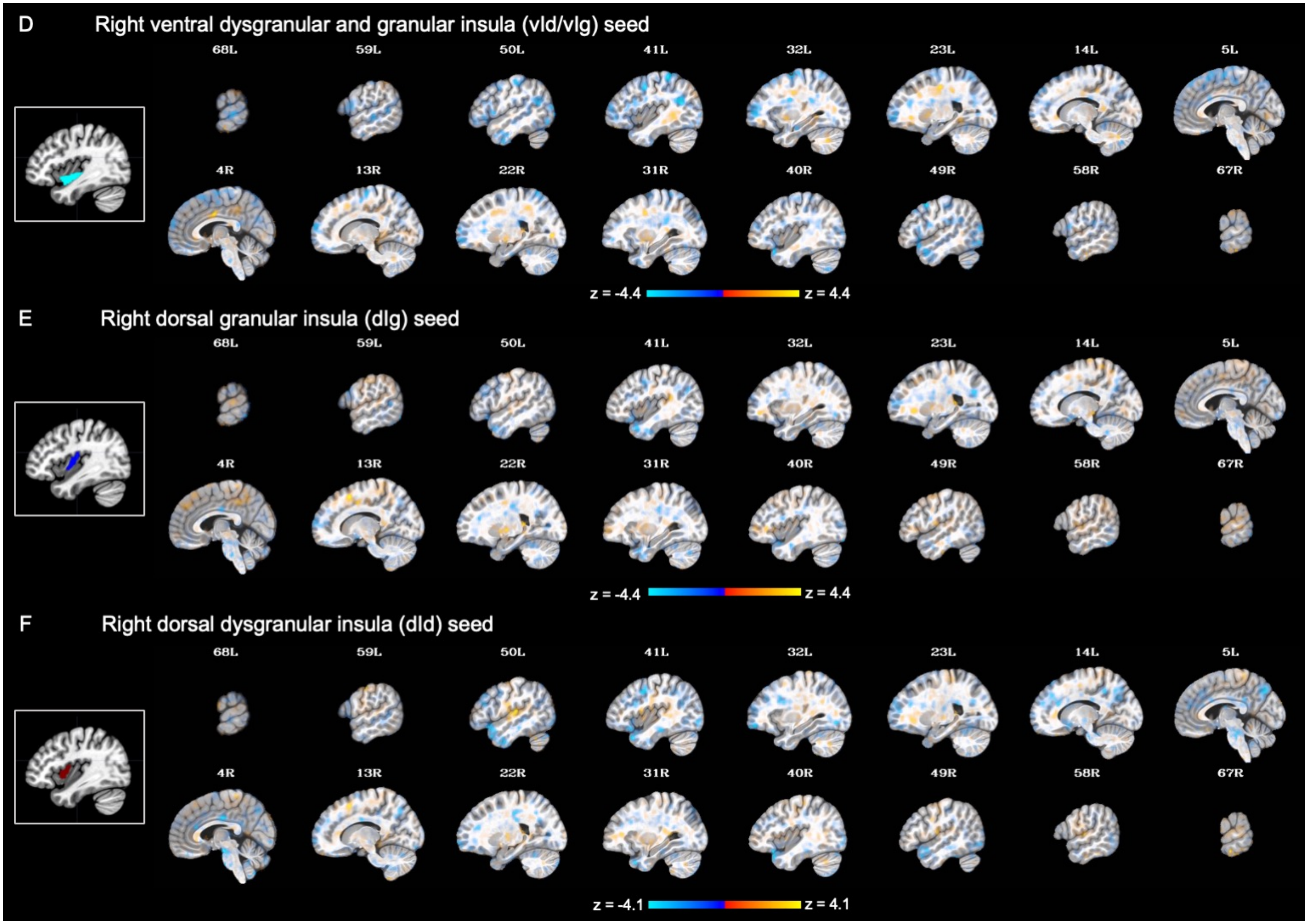

Note. A translucent statistical threshold set at $p < 0.001$ is applied, without cluster-extent correction. Areas exhibit increasing transparency corresponding to decreasing statistical significance (Taylor et al., 2023). Each seed region is displayed on the left side of its corresponding statistical map, framed with a white square outline.

Figure S4

Effect of diagnosis (MDD vs. HC) on seed-to-whole brain functional connectivity (left insula subregions as seeds).

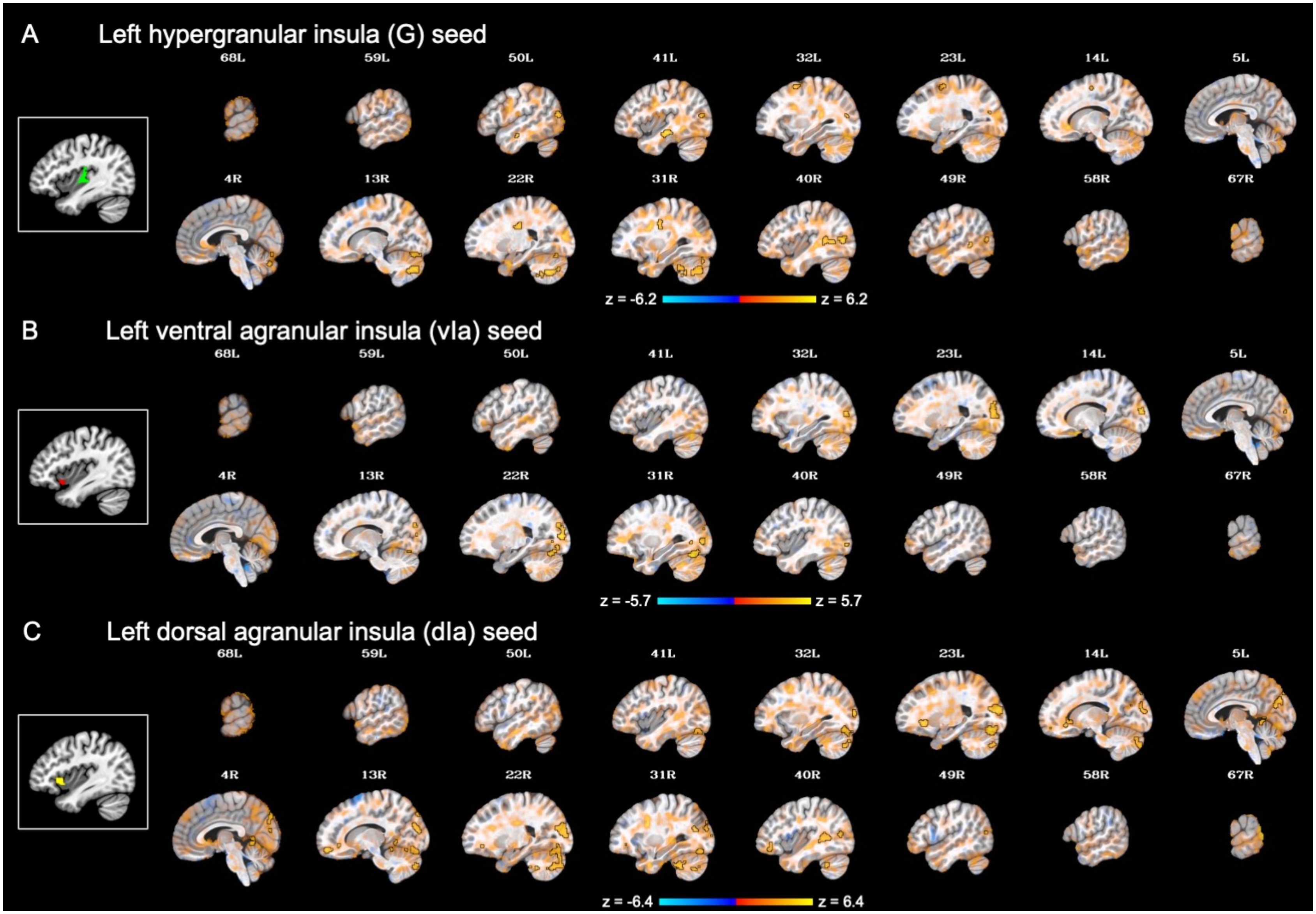

Figure S4 (Continued)

Effect of diagnosis (MDD vs. HC) on seed-to-whole brain functional connectivity (left insula subregions as seeds).

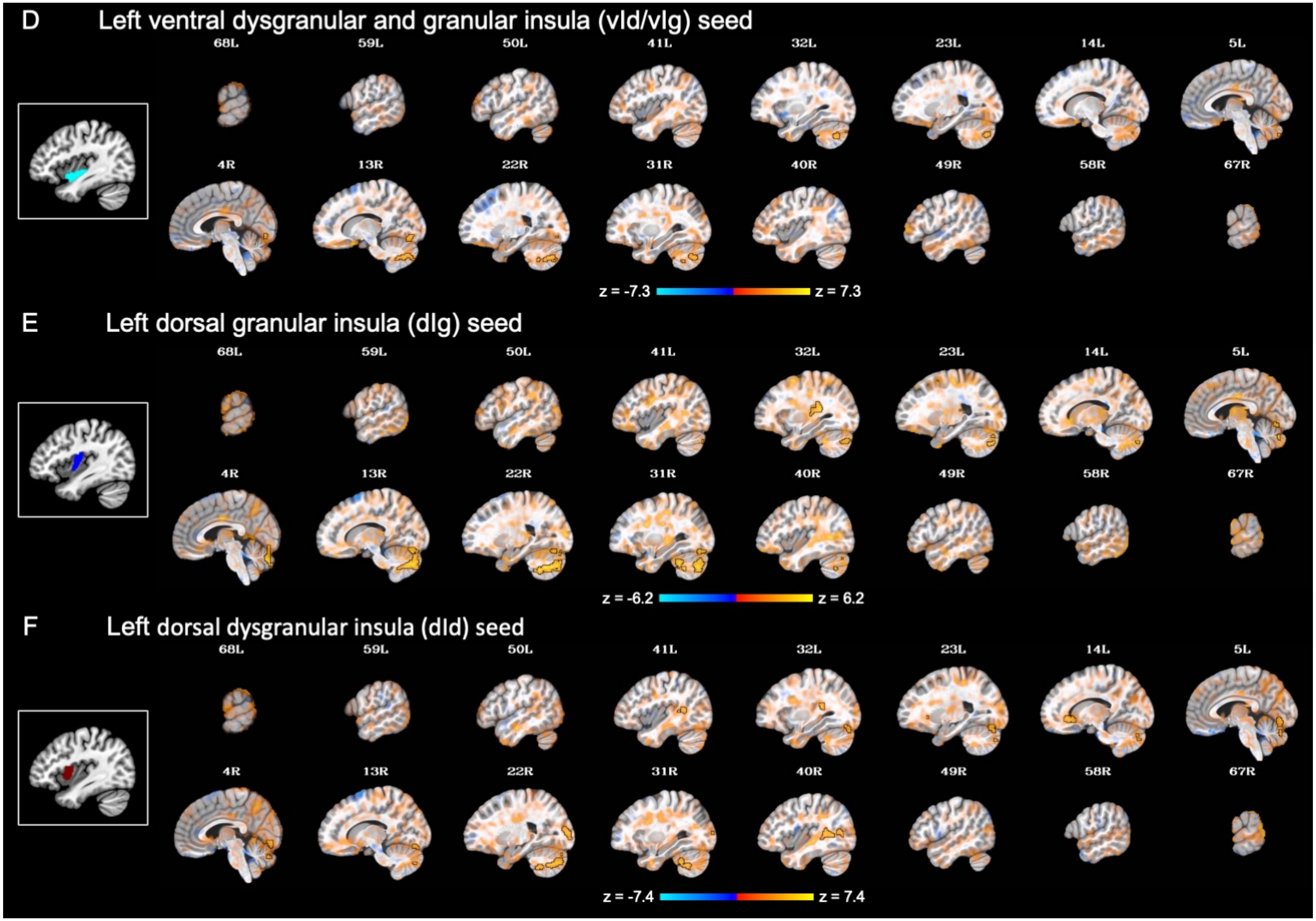

Note. A translucent statistical threshold is applied at $p < 0.001$, with cluster-extent correction at $k > 143$. Suprathreshold areas are emphasized with opacity and black outlines, while sub-threshold areas gradually become more transparent as their statistical significance decreases (Taylor et al., 2023).

Figure S5

Effect of diagnosis (MDD vs. HC) on seed-to-whole brain functional connectivity (right insula subregions as seeds).

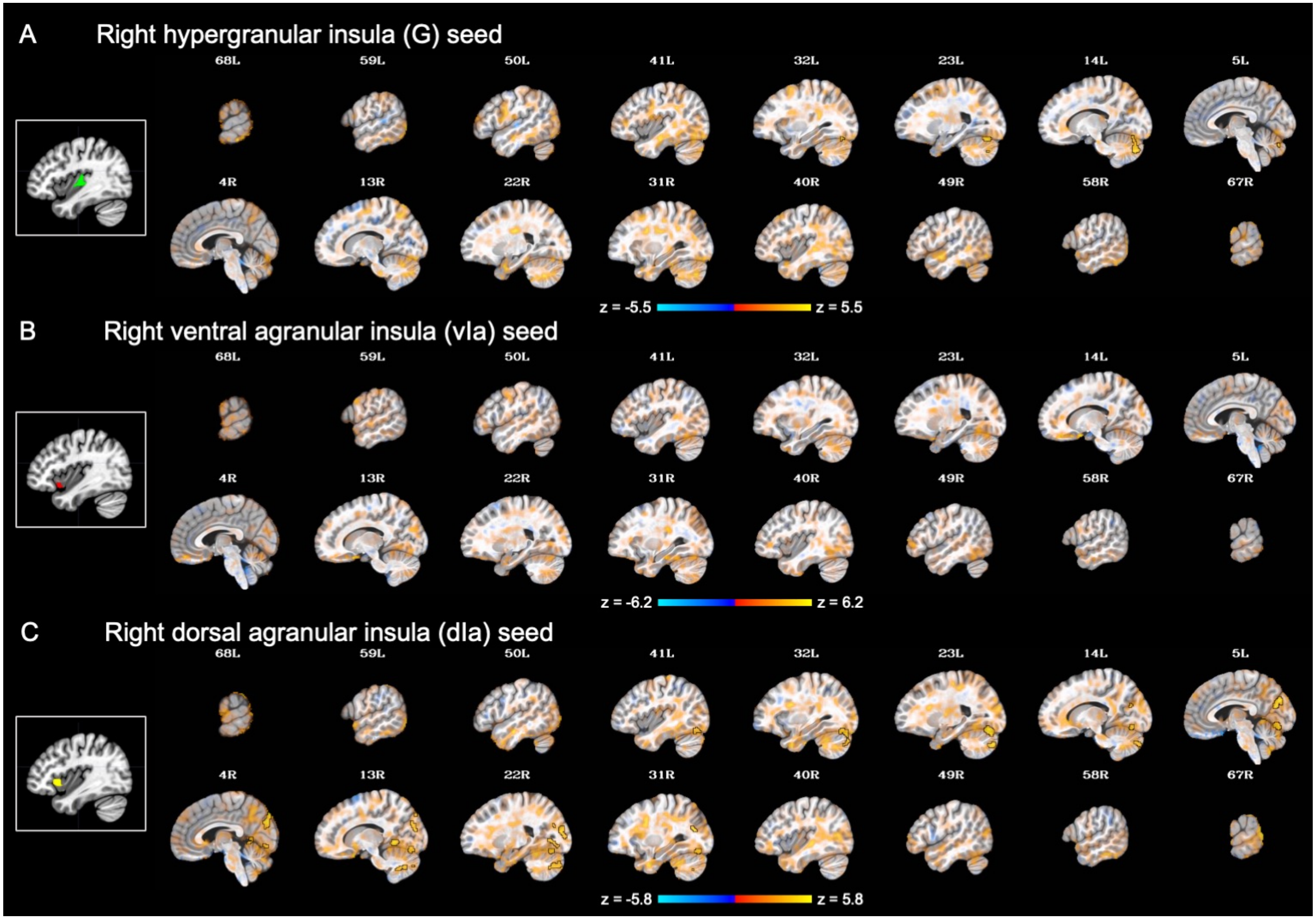

Figure S5 (Continued)

Effect of diagnosis (MDD vs. HC) on seed-to-whole brain functional connectivity (right insula subregions as seeds).

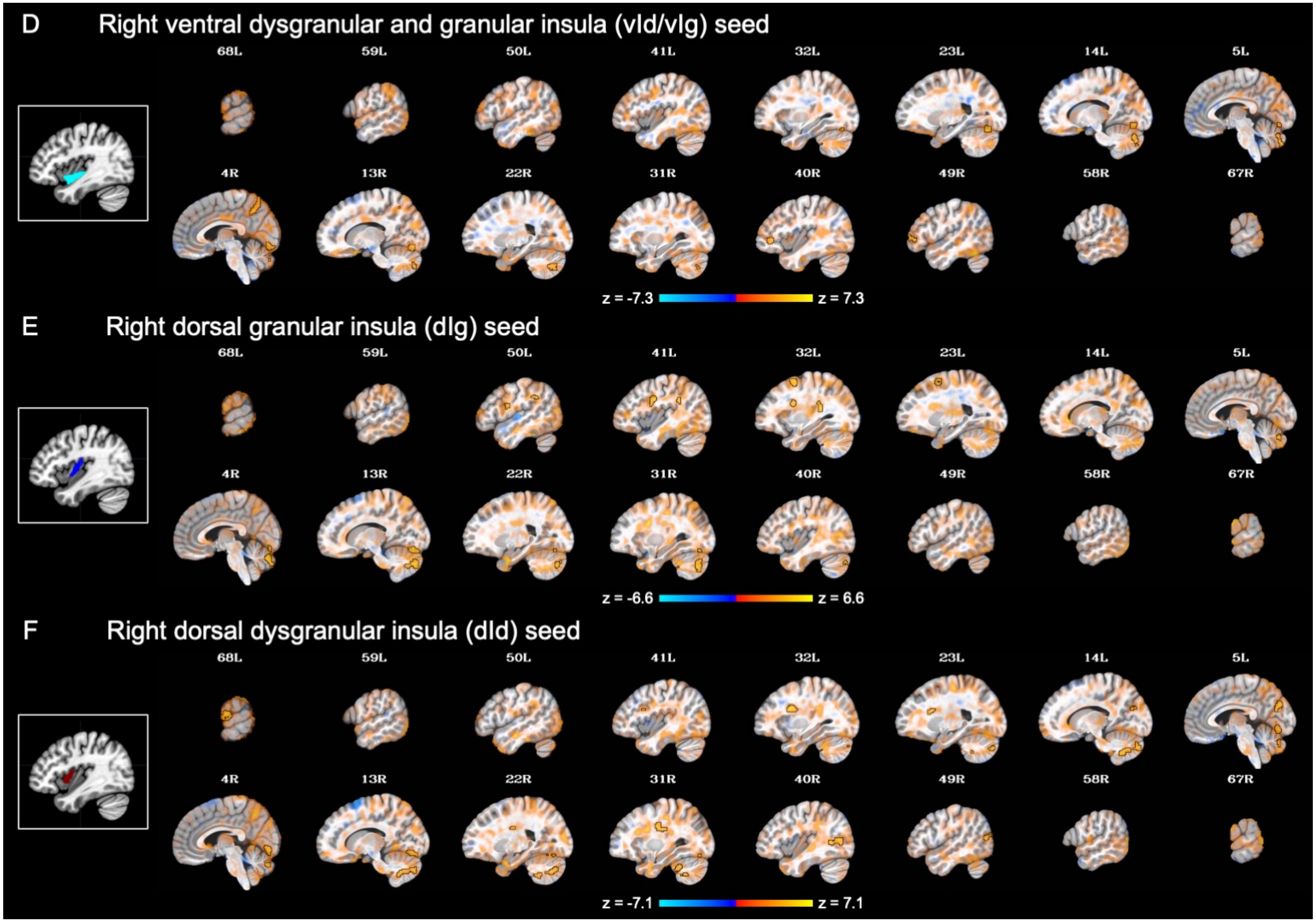

Note. A translucent statistical threshold is applied at $p < 0.001$, with cluster-extent correction at $k > 143$. Suprathreshold areas are emphasized with opacity and black outlines, while sub-threshold areas gradually become more transparent as their statistical significance decreases (Taylor et al., 2023).

Figure S6

Effect of run (RNT vs. Rest) on seed-to-whole brain functional connectivity (left insula subregions as seeds).

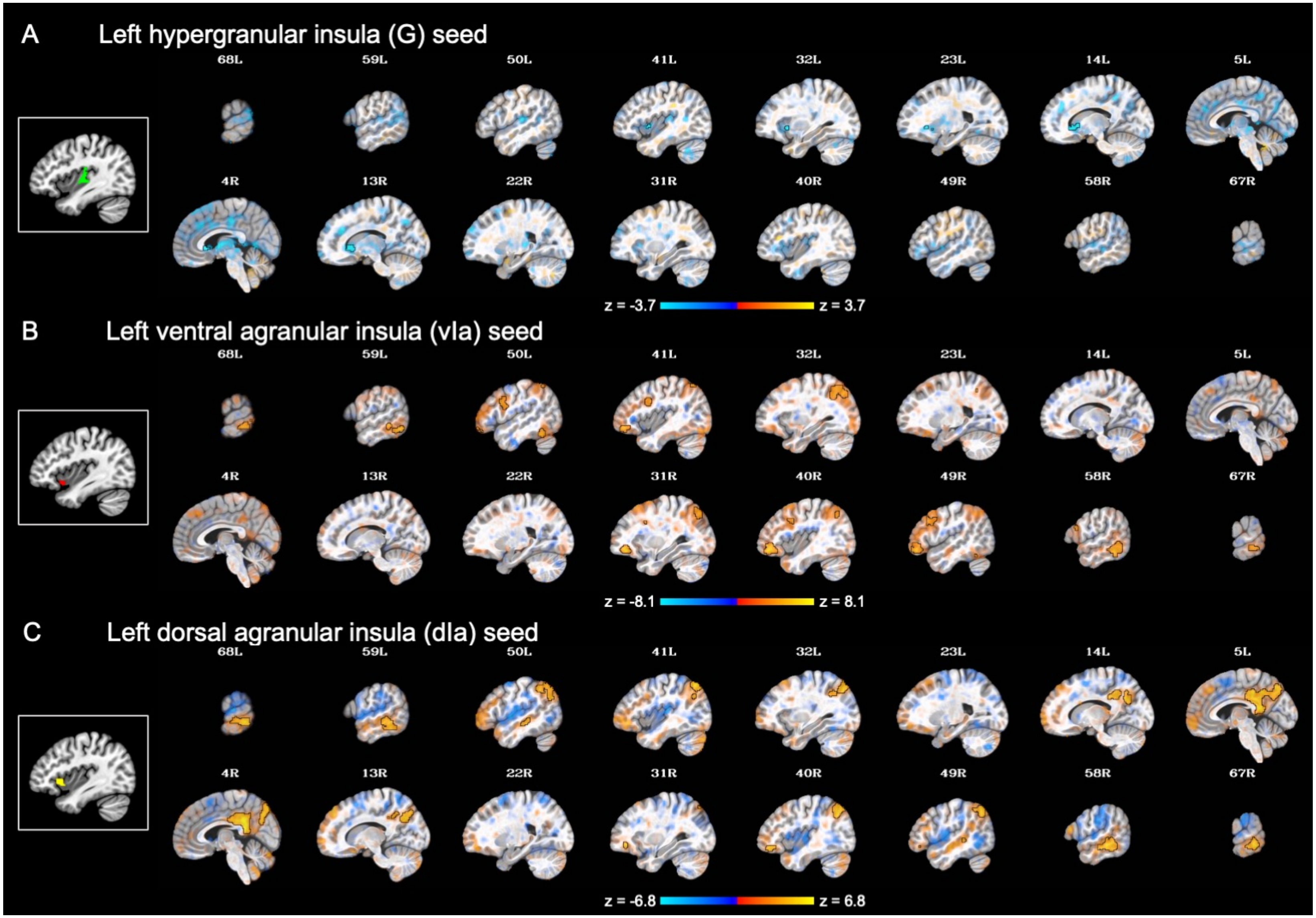

Figure S6 (Continued)

Effect of run (RNT vs. Rest) on seed-to-whole brain functional connectivity (left insula subregions as seeds).

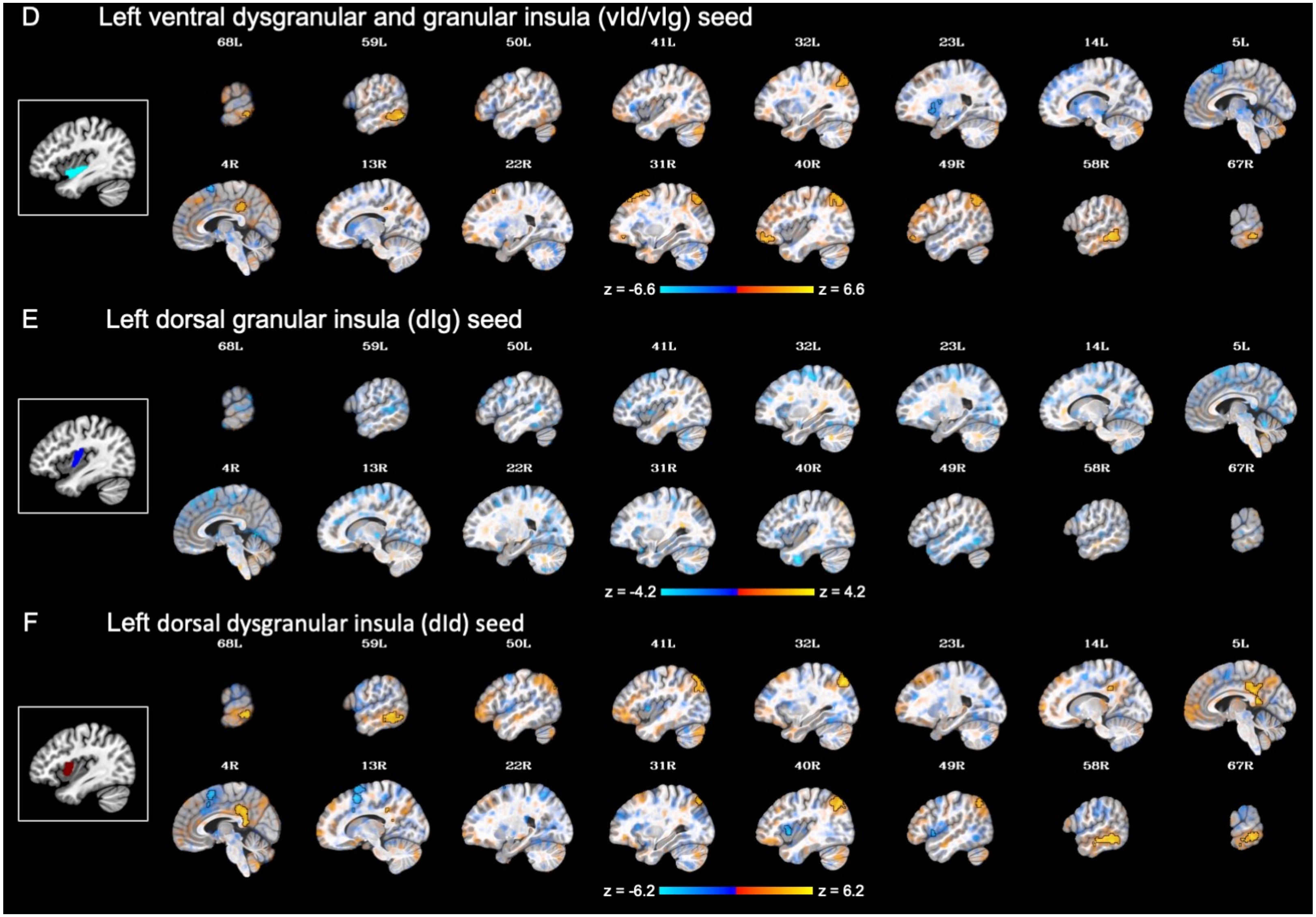

Note. A translucent statistical threshold is applied at $p < 0.001$, with cluster-extent correction at $k > 143$. Suprathreshold areas are emphasized with opacity and black outlines, while sub-threshold areas gradually become more transparent as their statistical significance decreases (Taylor et al., 2023).

Figure S7

Effect of run (RNT vs. Rest) on seed-to-whole brain functional connectivity (right insula subregions as seeds).

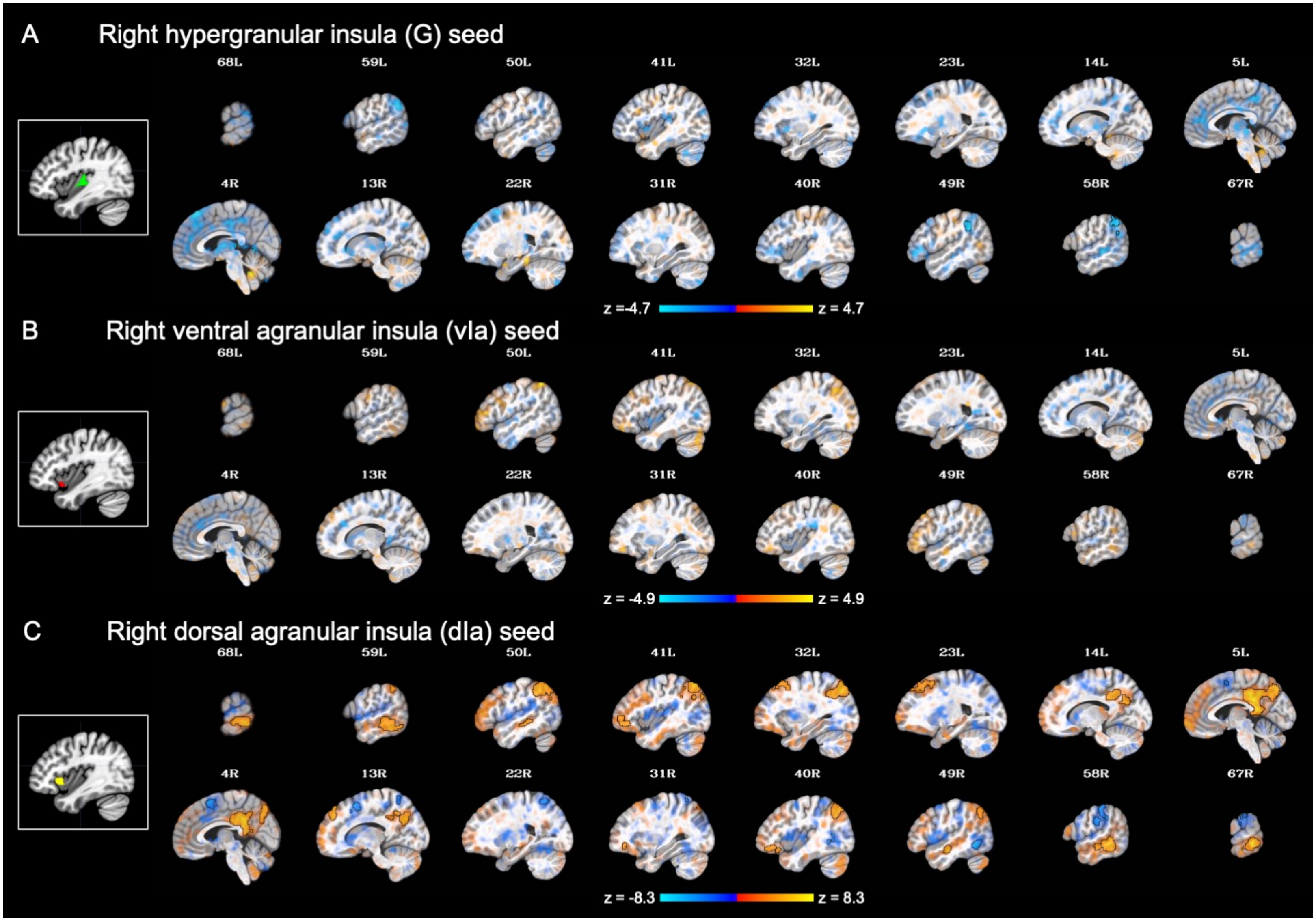

Figure S7 (Continued)

Effect of run (RNT vs. Rest) on seed-to-whole brain functional connectivity (right insula subregions as seeds).

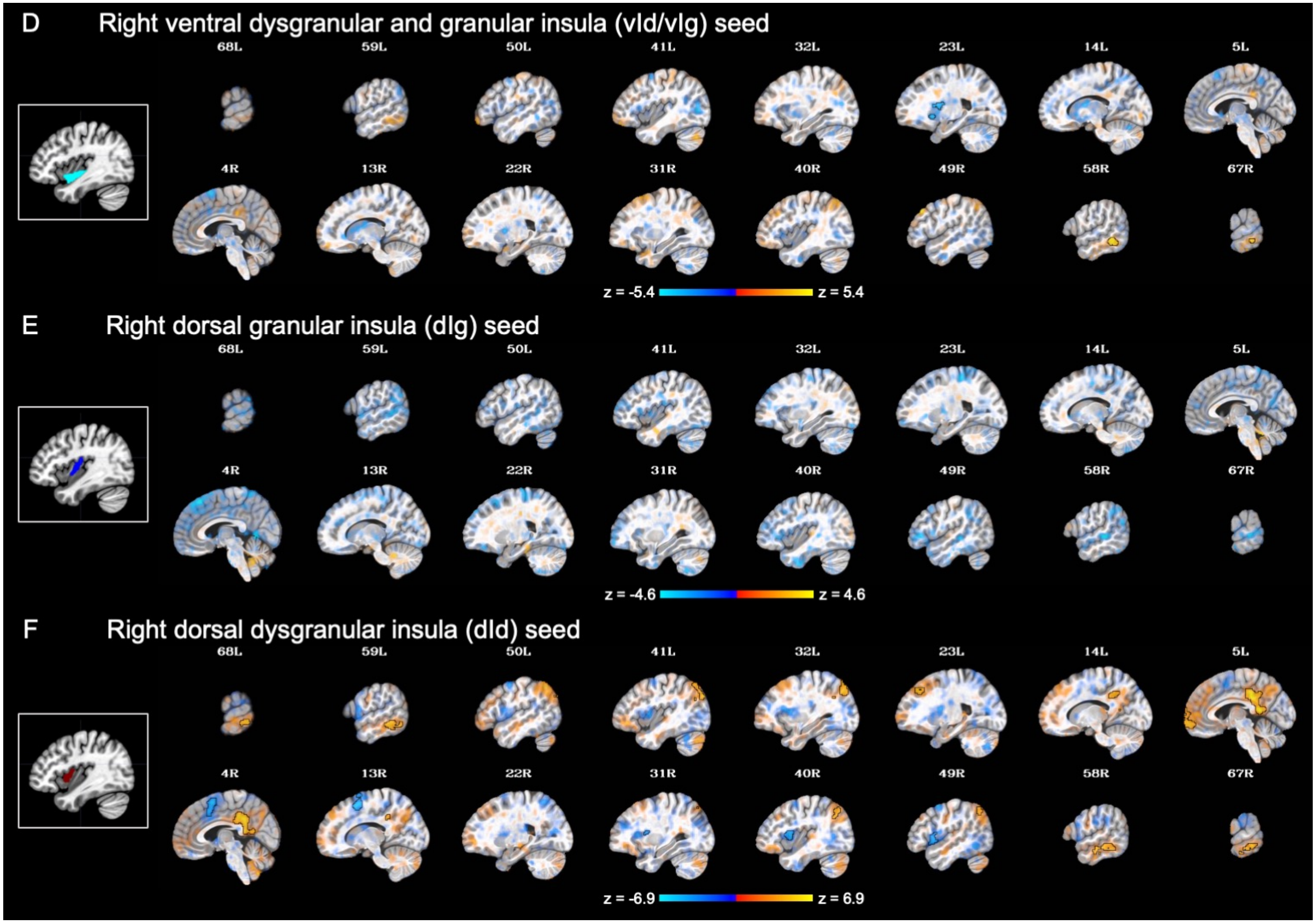

Note. A translucent statistical threshold is applied at $p < 0.001$, with cluster-extent correction at $k > 143$. Suprathreshold areas are emphasized with opacity and black outlines, while sub-threshold areas gradually become more transparent as their statistical significance decreases (Taylor et al., 2023).

References

Taylor, P. A., Reynolds, R. C., Calhoun, V., Gonzalez-Castillo, J., Handwerker, D. A., Bandettini, P. A., Mejia, A. F., & Chen, G. (2023). Highlight results, don't hide them: Enhance interpretation, reduce biases and improve reproducibility. *Neuroimage*, *274*, 120138. https://doi.org/10.1016/j.neuroimage.2023.120138
